# Nutrition Assessment of B-Vitamins in Highly Active and Sedentary Women

**DOI:** 10.3390/nu9040329

**Published:** 2017-03-26

**Authors:** Kathleen Woolf, Nicole L. Hahn, Megan M. Christensen, Amanda Carlson-Phillips, Christine M. Hansen

**Affiliations:** 1 Department of Nutrition and Food Studies, Steinhardt School of Culture, Education, and Human Development, New York University, 411 Lafayette, 5th Floor, New York, NY 10003, USA; 2 Department of Culinary and Nutrition Services, Banner Boswell Medical Center, 10401 W. Thunderbird Boulevard, Sun City, AZ 85351, USA; nicole.hahn@bannerhealth.com; 3 Department of Nutrition and Food Services, VA Salt Lake City Health Care System, 500 Foothill Drive, Salt Lake City, UT 84148, USA; megan.christensen@va.gov; 4 Department of Performance Innovation, Exos, 2629 E. Rose Garden Lane, Phoenix, AZ 85050, USA; acarlson@teamexos.com; 5 Nutrition Consultant, PO Box 184, Veneta, OR 97487, USA; veggiedoc@gmail.com

**Keywords:** B-vitamins, folate, vitamin B6, vitamin B12, female athlete

## Abstract

Background: Female athletes and active women require adequate nutrition for optimal health and performance. Nutrition assessments are needed to identify potential nutrients of concern. Folate, vitamin B6, and vitamin B12 function in important pathways used during physical activity and female athletes may be at risk for poor status of these micronutrients. This cross-sectional study described a comprehensive nutrition assessment of the B-vitamins (folate, vitamin B6, and vitamin B12) using both dietary (food and dietary supplements) and biochemical assessments among highly active and sedentary women. Methods: Highly active (*n* = 29; age 20 ± 2 years; body mass index (BMI) 23.8 ± 3.5 kg/m^2^) and sedentary (*n* = 29; age 24 ± 3 years; BMI 22.6 ± 3.0 kg/m^2^) women were recruited for this study. Participants completed 7-day weighed food records and a fasting blood draw. Results: Although the highly active women reported higher intakes of energy (*p* < 0.01), folate (*p* < 0.01), vitamin B6 (*p* < 0.01), and vitamin B12 (*p* < 0.01), no significant differences were found between the groups for biomarkers of folate, vitamin B6, and vitamin B12. All of the highly active women had biomarkers within the desired reference ranges, suggesting good status. In general, most participants were able to meet the 1998 Recommended Daily Allowance (RDA) from food alone. For the women that reported using dietary supplements, micronutrient intakes met the 1998 RDA and in some cases, exceeded the Tolerable Upper Intake Level. Conclusion: This nutrition assessment documented good status for folate, vitamin B6, and vitamin B12 in the highly active women. Similar assessment approaches (food, dietary supplements, and biomarkers) should to completed with other nutrients of concern for the female athlete.

## 1. Introduction

Female athletes and active women require adequate nutrition to stay healthy and perform optimally. Comprehensive nutrition assessments, including dietary, biochemical, anthropometric, clinical, and environmental components, are needed to identify specific nutrition-related problems that may impact overall health and performance. For example, the B-vitamins play important roles in maintaining the health of female athletes and active women, serving as coenzymes in pathways critical for physical activity [1,2,3,4,5,6]. Folate functions as a coenzyme in reactions of deoxyribonucleic acid (DNA) synthesis, red blood cell synthesis, and amino acid metabolism, including the conversion of homocysteine to methionine [4,7]. Pyridoxal 5’-phosphate (PLP), the biologically active form of vitamin B6 in the human body, serves as a coenzyme in transamination and deamination reactions of amino acid metabolism and activates the rate-limiting step of glycogen breakdown [4,8,9]. Vitamin B12 functions as a coenzyme in methyl transfer reactions (i.e., homocysteine to methionine) and helps recycle folate [4,10]. Vitamin B12 also assists with the breakdown of odd-numbered fatty acid chains, DNA synthesis, and the production of red blood cells. Because folate, vitamin B6, and vitamin B12 assist with the metabolism of homocysteine, plasma homocysteine concentrations increase without adequate supplies of folate, vitamin B6, and vitamin B12, leading to an increased risk of cardiovascular disease [11]. Thus, these B-vitamins aid in the utilization of energy, metabolism of amino acids, maintenance of red blood cells, and regeneration of tissue. Comprehensive nutrition assessment of these key micronutrients is crucial to an athlete’s success.

Unfortunately, many athletes, females in particular, may be at risk of poor dietary intakes for folate, vitamin B6, and vitamin B12 [12]. For instance, female athletes may not compensate for the energy expenditure associated with increased physical activity [13]. This behavior puts them at risk of low energy availability and many macronutrient and micronutrient deficits. In many sports, success is associated with a thin physique, thus encouraging excessive training and/or suboptimal dietary intakes [14,15]. Unfortunately, inadequate dietary intakes can impair an athlete’s performance and lead to fatigue, injury, and/or altered concentration [12]. Additionally, female athletes and active women may have less folate, vitamin B6, and vitamin B12 available for metabolism of homocysteine, potentially leading to elevated plasma homocysteine concentrations compared to their physically inactive peers.

Dietary assessment has been utilized to assess dietary intakes for folate, vitamin B6, and vitamin B12 and determine adequacy. Recent dietary intakes for the United States (US) adult population have been well summarized from the National Health and Examination Surveys (NHANES) [16]. For female athletes and active women, reported dietary intakes for these micronutrients tend to come from older studies and are challenging to interpret due to changes in the reference ranges used to define nutrient adequacy. When completing dietary assessment for folate, time of data collection should also be considered to account for the 1996 US mandatory fortification of enriched grain products with folic acid [17]. In 1998, the latest reference values for these micronutrients were published as part of the Dietary Reference Intakes (DRIs) [18]. The DRIs express nutrient adequacy as the Estimated Average Requirement (EAR) (representing 50% of the population’s requirement) and the Recommended Dietary Allowance (RDA) (representing 97.5% of the population’s requirement). However, the EAR may not be sufficient for a physically active adult, adding to the difficulty to make generalizations about nutrient adequacy. Notwithstanding these concerns, some studies report mean dietary intakes for folate in female athletes less than the 1998 EAR (320 µg/day DFE) [19,20,21,22,23,24]. However, more recent studies report higher dietary intakes for folate among female athletes [25,26], which may be a reflection of folic acid fortification. For vitamin B6, most studies in female athletes document adequate intakes when compared to the 1998 RDA (1.3 mg/day) and EAR (1.1 mg/day) [21,22,25,26]. Studies comparing dietary intakes of vitamin B6 to the 1980 RDA (2 mg/day for adult females) or 1989 RDA (1.6 mg/day for adult females), values much higher than the 1998 RDA, typically report inadequate mean intakes for female athletes [4,19,22,27]. Because vitamin B6 plays a major role in the metabolic pathways required during exercise (i.e., amino acid metabolism, gluconeogenesis, glycogenolysis), some research suggests that female athletes may require two to three times the 1998 RDA of vitamin B6 due to their increased physical activity patterns and protein requirements [4,8]. For vitamin B12, some studies report adequate dietary intakes in female athletes [19,20,21,25,26], while other studies report inadequate intakes [13,23].

Because of the risk of inadequate dietary intakes of folate, vitamin B6, and vitamin B12 in female athletes, some research has included biochemical assessment in the evaluation of nutrient status. Unfortunately, the results are quite mixed. For example, Matter et al. examined folate status in non-supplementing female marathon runners and reported that 33% had poor status evidenced by low serum folate concentrations [28]. However, Beals and Manore examined serum folate concentrations in female athletes and reported only 4% with poor folate status [29]. Approximately 50% of the female athletes in this study reported taking a dietary supplement. Other research has reported good folate status in female recreational athletes [26,30], runners [31], and endurance athletes [32]. Research has also examined vitamin B6 status in both male and female athletes, with equally mixed outcomes. For instance, Raczynski and Szczepanska assessed vitamin B6 status of elite male and female Polish athletes over 6 years using the erythrocyte alanine aminotransaminase activity coefficient, a functional measure of vitamin B6, and reported poor status in 9% of the athletes [33]. In this study, endurance athletes had the highest prevalence of poor status of vitamin B6 (13%). Poor vitamin B6 status was highest in the pre-Olympic years (16%) and lowest in Olympic years (3%), when athletes may have focused more on dietary intakes and dietary supplementation. More recently, Joubert and Manore reported good status in a study of 64 recreationally active athletes (38 female) using plasma PLP [26]. Most studies of vitamin B12 status in female athletes suggest the risk of poor status is low, when adequate energy and animal products are consumed. Although the research is much more limited, good vitamin B12 status has been reported in female ultra-marathoners [34] and recreationally active adults [26].

More research should include comprehensive nutrition assessments to examine B-vitamin status in female athletes, especially for folate, vitamin B6, and vitamin B12. When completing dietary assessments, previous research has not included the contribution of natural sources and synthetic sources (fortified foods, dietary supplements) to dietary intakes. To determine nutrient status, biochemical assessment should also be included. Unfortunately, mixed gender studies have included more male participants than female participants, limiting information on the B-vitamin status of the female athlete. Thus, the purpose of this study was to describe the approach and results of a comprehensive nutrition assessment for B-vitamins (folate, vitamin B6, and vitamin B12), including dietary (food and dietary supplements) and biochemical assessments, among highly active and sedentary women.

## 2. Methods

### 2.1. Participant Recruitment and Study Design

This cross-sectional study completed a nutrition assessment of the B-vitamins (folate, vitamin B6, and vitamin B12) among highly active and sedentary women. This study was approved by the Institutional Review Board (IRB) at Arizona State University (IRB #0511000343; initial approval date 15 December 2005) and the University Committee on Activities Involving Human Subjects at New York University (IRB #11-8778; initial approval date 9 January 2012) and was conducted according to these guidelines.

Highly active and sedentary women between 18 and 35 years of age were recruited as the research participants for this study. Recruitment flyers were posted at university and college campuses, athletic training facilities, community centers, libraries, and throughout the local community. The study investigators also sent recruitment flyers to collegiate teams. The recruitment flyers briefly described the study and invited women to contact the study investigators for more information.

The study investigators determined eligibility over the telephone based on the following criteria: age (between 18 and 35 years), weight stable (<10% weight loss or gain within the past 6 months), no pregnancy or breastfeeding within the past year, nonsmoker or limited social smoker (quit smoking at least 6 months prior to study entry or smoke a few cigarettes socially on one occasion and then not smoke again for several days or weeks), and activity (highly active group defined as engaging in ≥12 h per week of programmed physical activity; sedentary group defined as engaging in <2 h of programmed physical activity per week). These activity levels must have been maintained for at least a year prior to study participation. Women who met the study criteria were invited to schedule a study appointment.

### 2.2. Procedures

The study participants completed two study visits. During the first visit, participants received detailed information about the study and signed an informed consent form. Height and weight were measured and body mass index (BMI) was calculated for each study participant. Participants were interviewed about the use of medications (prescription and over-the-counter) and dietary supplements (i.e., protein, energy, carbohydrate, meal replacement, vitamin, mineral, or herbal) and completed a health history questionnaire. Participants were asked to keep a 7-day weighed food record, noting all foods, beverages, and dietary supplements consumed. The study investigators provided participants with a food scale (Metrokane Gourmet Weigh Scale, Metrokane, New York, NY, USA) and showed them how to weigh foods. Participants were encouraged to include food labels for packaged items and provide measurements in teaspoons, tablespoons, or cups for foods not able to be weighed.

A second study visit was scheduled after the participants completed the food records. At this visit, participants completed an eight-hour fasting blood draw to determine blood biomarkers of folate, vitamin B6, and vitamin B12. The food records and study questionnaires were reviewed for completeness and study supplies were retrieved.

### 2.3. Anthropometric Assessment

Height was determined using a portable stadiometer (Invicta Plastics Limited, Oadby, Leicester, UK) to the nearest 0.1 centimeter without shoes. A Seca Bella 840 electronic flat scale (Seca North America, Chino, CA, USA) obtained each participant’s weight to the nearest 0.1 kilogram.

### 2.4. Dietary and Physical Activity Assessment

Participants completed a 7-day weighed food record to examine dietary intake of energy, folate, vitamin B6, and vitamin B12. At the end of each day, participants recorded the type and duration of any programmed physical activity completed. The food records were analyzed using Food Processor, version 8.5 (Esha Research, Salem, OR, USA) and the United States Department of Agriculture (USDA) National Nutrient Database for Standard Reference, Release 20. The USDA database provided additional micronutrient information for commercial products. Folate intakes included assessments of dietary food folate (natural), synthetic folic acid added to fortified foods, and dietary folate equivalents (DFE) ((synthetic folic acid × 1.7) + food folate (natural)). The intake total included both natural and synthetic forms of vitamin B12. For vitamin B6, the intake total included vitamin B6 from food. Dietary intakes over the 7-days were averaged to determine the reported daily intake.

Micronutrient intakes (folic acid, vitamin B6, and vitamin B12) from supplements were added to the totals from food for those participants that reported using dietary supplements. The amount of folic acid from supplements was multiplied by 1.7 before adding to the total from food. Intakes from dietary supplements were summarized to reflect reported average daily intakes, considering dosage and usage patterns (days/week or days/month).

The estimated energy requirement (EER) was calculated for each participant using the appropriate age and gender equation [35]. For the active women, the “very active” physical activity coefficient was used in the equation. For the sedentary women, the “sedentary” physical activity coefficient was used. Energy intake/EER was determined for each participant.

### 2.5. Biochemical Assessment

Participants completed an eight-hour fasting blood draw to determine concentrations of plasma folate, red blood cell folate, plasma vitamin B6, vitamin B12, transcobalamin II, and homocysteine. Blood samples were immediately placed on ice and centrifuged within 30 min at 3000× *g* for 10 min at 4 °C. After centrifugation, the blood was separated and the plasma samples stored at −44 °C until analysis. Sonora Quest (Phoenix, AZ, USA), an independent laboratory, determined mean cell volume, hemoglobin, hematocrit, and high sensitivity C-reactive protein (CRP) concentrations.

For the analysis of red blood cell folate, a whole blood dilution (1:21) was prepared by combining 100 μL of well-suspended blood to 2 mL of newly made 0.2% ascorbic acid solution. The diluted samples were wrapped in foil to prevent light penetration and stored at −44 °C until the time of analysis. Plasma folate and red blood cell concentrations were analyzed using the Becton Dickinson SimulTRAC^®^-S Solid Phase Radioassay Kit (Becton, Dickinson and Company, Franklin Lakes, NJ, USA) for Vitamin B12 (^57^Co) and Folate (^125^I).

High performance liquid chromatography (HPLC) was used to determine vitamin B6 status of the participants using PLP as the biomarker [36]. HPLC utilizes reverse-phase ion pairing to separate B6 vitamers, which are chromatographically measured at an excitation wavelength of 330 nm and fluorescent emission of 400 nm.

Vitamin B12 status was assessed using plasma vitamin B12 and holotranscobalamin II (transcobalamin II) concentrations [37]. Transcobalamin II represents newly absorbed vitamin B12 enroute to the hematopoietic system and proliferating cells and is a more sensitive indicator of vitamin B12 status than plasma vitamin B12. Transcobalamin II was assessed by first preparing a slurry that contained 3 g synthetic amorphous precipitated silica in 20 mL of deionized water [38,39]. Transcobalamin II was absorbed from the samples by adding 100 μL of the prepared slurry to 500 μL of plasma and letting the samples sit at room temperature for 10 min. The samples were then centrifuged at 5000× *g* for 10 min. The supernatant was retained for further analysis. The Becton Dickinson SimulTRAC^®^-S Solid Phase Radioassay Kit (Becton, Dickinson and Company, Franklin Lakes, NJ, USA) for Vitamin B12 (^57^Co) and Folate (^125^I) was used to measure plasma vitamin B12 and holo-haptocorrin concentrations. Transcolbalamin II concentrations were determined by subtracting the holo-haptocorrin concentrations from the total plasma vitamin B12 concentration.

Fasting plasma homocysteine, a functional biomarker of folate, vitamin B6, and vitamin B12 status, was also measured by HPLC with fluorescence [40,41].

### 2.6. Statistical Analysis

Power calculations were completed using the reported vitamin B6 and folate dietary intakes for athletes and sedentary individuals from the research literature [42,43]. Using a difference of 0.2 mg, a sample size of 15 women per group would be sufficient to detect a difference in reported vitamin B6 intake between groups with a power of 0.80 and α = 0.05. However, a sample size of 57 women per group would be required to detect a difference in reported folate intake (using a difference of 50 μg) between groups with a power of 0.80 and α = 0.05, beyond the reach of this pilot study. Thus, we aimed to have 30 women per group and recruited additional women to allow for attrition. Prior to the statistical analysis, the data were tested for normality. Histograms of the study outcome measures visually assessed the distribution of the data. The Kolmogorov-Smirnov statistic was also used to assess normality of the distribution scores. Descriptive statistics (mean and standard deviation) were determined for the demographic data for the two groups of women. Independent sample *t*-tests compared the outcome measures between groups for the normally distributed variables. Because CRP did not have a normal distribution, the Mann-Whitney U test was used to examine the differences between groups. The median and interquartile range were used to summarize these values. Data were analyzed using IBM Statistical Package for the Social Sciences (SPSS) Statistics for Windows, version 22.0 (IBM Corporation, Armonk, NY, USA) version 14.0 and determined to be significant if *p* < 0.05.

## 3. Results

### 3.1. Descriptive Characteristics

Seventy-five participants signed consent forms (41 highly active women and 34 sedentary women). However, 11 highly active and 3 sedentary women decided not to finish the study. One highly active woman was eliminated because she was not as active as previously reported. Two sedentary women were eliminated; one had a blood disorder, and one had an activity level that was greater than 2 h per day. The present analysis includes 29 highly active women and 29 sedentary women.

Table 1 summarizes the descriptive characteristics for the 58 participants. The sedentary women were older than the highly active women (*p* < 0.01). Although the highly active women were heavier than the sedentary women, height and BMI were not significantly different between the two groups of women. The majority of participants in both groups, 69% of the highly active women and 82% percent of the sedentary women, reported their race/ethnicity as Caucasian (not of Hispanic origin). The highly active women reported consuming more total energy (*p* < 0.01) and relative energy (kcal/kg body weight) (*p* = 0.01) than the sedentary women. Although EER was significantly greater in the highly active women compared to the sedentary women (*p* < 0.01), there were no differences between groups for percent energy intake/EER.

Table 1 also describes the highly active women by their sport. The highly active women consisted of student athletes from Division I, Division II, and community college athletic teams.

### 3.2. Dietary Assessment

Table 2, Table 3, Table 4, Table 5 and Table 6 summarize the reported micronutrient intake of the 58 highly active and sedentary women. Folate intakes from food (natural sources, fortified foods) and dietary supplements are summarized in Table 2. The highly active women reported a greater intake of natural folate (μg/day) (*p* < 0.01), folic acid from fortified foods (μg/day) (*p* = 0.03), and folate (natural + fortified foods) (*μ*g/day dietary folate equivalents (DFE)) (*p* < 0.01) than the sedentary women. There were no differences between the two groups of women for folate density (μg/day DFE/1000 kcal). For those participants that reported dietary supplement use (highly active = 9; sedentary = 12), the sedentary women consumed more folic acid from dietary supplements (μg/day) (*p* = 0.04) than the active women. However, there were no additional differences in folate intakes between the groups for participants that reported using dietary supplements.

Table 3 outlines the vitamin B6 intake from food and dietary supplements for the 58 highly active and sedentary women. Significant differences between groups were found in vitamin B6 intake from food (mg/day) (*p* < 0.01) and vitamin B6 density from food (mg/1000 kcal) ( *p* = 0.03). Eight highly active and 12 sedentary women reported the use of supplements containing vitamin B6. However, there were no significant differences between groups for vitamin B6 intakes for those participants that used dietary supplements.

Dietary and supplemental intake of vitamin B12 is summarized in Table 4. The highly active women reported a significantly higher intake of vitamin B12 from food (μg/day) (*p* < 0.01) than the sedentary women. No significant differences were found between groups for synthetic vitamin B12 added to food (μg/day) and vitamin B12 density (μg/1000 kcal) from food. Nine highly active women reported the use of supplements containing vitamin B12 compared to 12 sedentary participants. However, there were no significant differences between groups for vitamin B12 intakes for those participants that reported using dietary supplements.

Table 5 describes the adequacy of dietary micronutrient intake as set by the DRI recommendations for folate, vitamin B6, and vitamin B12. Ninety-six percent of the highly active women and 90% of the sedentary participants met the EAR for folate. Ninety percent of the highly active women and 79% of the sedentary women met the RDA for folate. Fourteen percent of the highly active women and 3% of the sedentary women exceeded the UL (Tolerable Upper Intake Level) for folate, a guideline that only applies to synthetic forms of the nutrient (i.e., fortified foods, dietary supplements). Thus, folic acid from fortified foods was the form of folate used in this estimation. For vitamin B6, 100% of the highly active women and 90% of the sedentary women met the EAR. Ninety-three percent of the highly active women and 86% of the sedentary women met the RDA for vitamin B6. The UL was not exceeded by either group of women for vitamin B6. For vitamin B12, 100% of the highly active women met the EAR and RDA. One hundred percent of the sedentary women met the EAR for vitamin B12 and 93% of the sedentary women met the RDA. Currently, a UL for vitamin B12 has not been established.

Table 6 describes the adequacy of dietary micronutrient intake from food and dietary supplements as set by the DRI recommendations for folate, vitamin B6, and vitamin B12 for those participants reporting dietary supplement use. All of the highly active and sedentary participants met the EAR and RDA for folate, vitamin B6, and vitamin B12 when including both food and dietary supplements. However, 5 of the highly active (56% of those reporting dietary supplement use) and 8 of the sedentary women (67% of those reporting dietary supplement use) exceeded the UL for folate, a guideline referring to synthetic folate sources (i.e., fortified foods, dietary supplements). None of the highly active woman exceeded the UL for vitamin B6, whereas 1 sedentary woman did.

### 3.3. Blood Biochemical Assessment

Table 7 summarizes biomarkers of B-vitamin status for the two groups of women. No differences were found between groups for any of the biomarkers for folate, vitamin B6, and vitamin B12. None of the participants had a plasma folate, red blood cell folate, or plasma vitamin B12 concentration below the reference range. However, two (7%) of the sedentary participants had plasma transcobalamin II concentrations below the reference range. Two (7%) of the highly active women and two (7%) of the sedentary participants had plasma transcobalamin II concentrations above the reference range. Additionally, two (7%) of the sedentary participants had PLP concentrations below the reference range.

Table 7 also describes the hematological data for the study participants. One athlete had a mean corpuscular volume (MCV), hemoglobin, and hematocrit values below the reference range. No participant was found to have an MCV value above the reference range. However, there were no significant differences between groups for these three hematological markers and CRP. Thirty-four percent of the athletes and 28% of the sedentary participants had CRP values above the reference value.

## 4. Discussion

This study is one of few that summarizes a comprehensive nutrition assessment of the B-vitamins using dietary (food and dietary supplements) and biochemical assessments in highly active women compared to a control group of sedentary women. As part of the dietary assessment, food records were collected over 7 days (longer than other studies in the research literature) to determine nutrient adequacy using the DRIs for folate, vitamin B6, and vitamin B12. The average micronutrient intake of the highly active and sedentary participants not only met the 1998 DRIs, but were much higher than the dietary intakes reported in previous studies. Additionally, four highly active women and one sedentary participant exceeded the UL for folate with the consumption of fortified foods. Information was collected on dietary supplement use as part of the dietary assessment. The participants that used dietary supplements met the 1998 RDA for folate, vitamin B6, and vitamin B12. Furthermore, five highly active and eight sedentary women exceeded the UL for folate when the intake included both food and supplements. Dietary supplement use has not always been reported or included in previous studies. As part of the biochemical assessment, biomarkers for folate, vitamin B6, and vitamin B12 were determined. The mean values for the biomarkers were not significantly different between the two groups of women. All of the highly active women had biomarkers for folate, vitamin B6, and vitamin B12 within the reference ranges, suggesting good status. However, two sedentary women had low transcobalamin II concentrations, suggesting poor status of vitamin B12, and two different sedentary women had low PLP concentrations, suggesting poor status of vitamin B6.

### 4.1. Dietary Assessment

Folate, vitamin B6, and vitamin B12 intakes from food were significantly higher in the highly active women compared to the sedentary women. This finding may be related to the significantly higher energy intake by the highly active women. Only vitamin B6 was significantly different between groups for nutrient density, with a higher density in the highly active women; however, nutrient density recommendations were met for all three nutrients by both groups. Thus, the highly active women were not necessarily consuming more nutrient dense foods, especially for folate and vitamin B12. Athletes with lower energy requirements may benefit from nutrition education to help them select more nutritious foods in the diet. The average dietary intake of vitamin B6, folate, and vitamin B12 for the women in our study exceeded the 1998 RDAs for folate (400 μg/day of DFE), vitamin B6 (1.2 mg/day for girls 14–18 years; 1.3 mg/day for women 19–50 years), and vitamin B12 (2.4 μg/day). The reported dietary intakes from this study are also much higher than the intakes reported in recent NHANES data (i.e., women 20–29 years of age: vitamin B6 = 1.91 mg, folate = 471 μg DFE, vitamin B12 = 4.23 μg) [16].

In our study, the dietary intakes for folate are higher than those reported by female athletes in previous studies conducted after the mandatory folic acid fortification [13,14,23,26]. For example, 25 synchronized figure skaters reported average intakes for folate of 65% of the 1998 RDA [14]. In another study, pre- and post-season intakes for folate were examined in 13 intercollegiate female soccer players [13]. Pre-season intakes were 271 ± 130 μg/day, while the post-season intake was reported as 186 ± 113 μg/day. Similarly, Leydon and Wall examined dietary intakes among female jockeys and reported average dietary intakes of only 132 ± 52 μg/day [23]. Among female recreational athletes, Joubert and Manore reported higher dietary intakes for folate of 428 ± 125 μg/day for female athletes participating primarily in low intensity activities and 511 ± 105 μg/day for female athletes participating in high intensity activities [26].

Similarly, the reported vitamin B6 intakes in our study are higher than those reported by female athletes in the research literature [13,14,23,26]. Among female soccer players, the mean pre-season vitamin B6 intake met the 1998 RDA, but the mean post-season intake did not [13]. The post-season overall energy intake was less than the pre-season intake and could account for a decreased vitamin B6 intake. Ziegler et al. examined the vitamin B6 intake of female synchronized figure skaters utilizing 3-day food records of 123 athletes and reported results by age [14]. The mean values for all participants and the girls aged 14–18 years did not meet the 1998 RDA for vitamin B6. The lower intakes reported in this study may be due to lower energy intakes as figure skating is seen as a weight conscious sport. For female jockeys, another weight conscious sport, the average vitamin B6 intakes were 0.90 ± 0.49 mg/day, less than the 1998 RDA and EAR [23]. However, other research has reported adequate vitamin B6 intakes. For instance, Joubert and Manore reported mean vitamin B6 intakes from 2.2 ± 1.6 to 2.4 ± 0.7 mg/day in recreational athletes, above the current RDA of 1.3 mg/day for women 19–50 years of age [26].

In the research literature, the mean intakes of vitamin B12 for female athletes are lower than those found in our study [13,14,23,26]. For example, the average pre- and post-season vitamin B12 intakes of female soccer players were 4.5 ± 1.9 μg/day and 2.1 ± 1.7 μg/day, respectively [13]. The pre-season intake was also higher in dietary protein, which possibly influenced the average vitamin B12 intake. The post-season average intake did not meet the 1998 RDA, but was adequate when compared to the EAR, a guideline that may not be appropriate for an athlete. In female synchronized figure skaters, the average intake met the 1998 RDA [14]. However, younger skaters (14–18 years) had average intakes of 2.2 μg/day for vitamin B12, less than the RDA. Leydon and Wall reported dietary intakes of vitamin B12 less than the RDA among female jockeys (2.15 ± 1.07 μg/day) [23]. Joubert and Manore reported dietary intakes for vitamin B12 that exceeded the RDA among female recreational athletes (5.3 ± 2.5 to 5.3 ± 4.8 μg/day) [26]. Our highly active women reported much higher vitamin B12 intakes (active = 8.1 ± 6.3 μg/day; sedentary = 4.7 ± 2.4 μg/day), which may be attributed to a more complete dietary assessment, utilizing a dietary software database that also included synthetic food sources of vitamin B12.

The current study reported higher intakes of vitamin B6, folate, and vitamin B12 than most other studies. Several factors may have contributed to this finding. First, when the dietary software program was missing micronutrient information for the B vitamins, we used the USDA nutrient database to reanalyze the food records. The USDA nutrient database was more complete in regards to B-vitamin content, including synthetic forms of folate and vitamin B12. Other studies may have used software with similar missing data, thus, leading to an underestimation of dietary intakes. Second, our participants reported average intakes of 867 ± 391 μg/day (active) and 595 ± 250 μg/day (sedentary) DFE for folate. Food records showed large quantities of ready-to-eat breakfast cereals consumed by both groups of women. Some participants consumed >3 cups per sitting with multiple sittings per day. Four (14%) of the highly-active and one (3%) of the sedentary women had folate intakes that exceeded the UL of 1000 μg/day. Among US adults, Yang et al. reported that 2.7% of adults consumed more than the UL of folic acid, a percentage similar to the sedentary women in our study [45]. Thus, the consumption of ready-to-eat cereal was most likely associated with the higher dietary intakes of folic acid.

Two studies in female athletes also included dietary supplements when completing a dietary assessment of B-vitamins [25,34]. Singh et al. examined dietary intakes of ultra-marathon runners and found the average intake of vitamin B6 from food was 2.6 ± 0.3 mg/day for the usual diet and 2.3 ± 0.3 mg/day for the pre-race diet [34]. Vitamin B6 intake jumped to 7.3 ± 2.2 mg/day (usual diet) and 7.0 ± 2.4 mg/day (pre-race diet) when food and supplement intakes were combined. Average dietary folate intakes of the ultra-marathon runners were 391 μg/day, which only met the 1998 EAR of 320 μg/day of DFE [34]. When supplemental folic acid was included, average intakes of dietary folate increased (629 ±102 μg/day for usual intake and 513 ± 92 μg/day for pre-race intake). Singh et al. reported the average intake of vitamin B12 from food as 6.1 μg/day for the usual diet and 4.5 μg/day for the pre-race diet from food. Inclusion of supplemental vitamin B12 intake increased the total intake to 51.3 μg/day (usual diet) and 51.8 μg/day (pre-race diet), well above the 1998 RDA. Beshgetoor and Nichols also described the food and supplement intake of 25 female master cyclists and runners [25]. The mean intake of vitamin B6 for the supplementing athletes (SA) and the non-supplementing athletes (NSA) was 15 ± 5 mg/day and 3 ± 1 mg/day, respectively. Both groups met the 1998 RDA. The mean intake of folate for the SA and NSA was 486 ± 55 and 402 ± 115 μg/day DFE, respectively. The mean vitamin B12 intake for the SA and NSA was 18 ± 5 and 6 ± 2 μg/day, respectively, well above the EAR and RDA. Similarly, our study also documented higher consumption of folate, vitamin B6, and vitamin B12 for the highly active and sedentary women when both food and dietary supplements were included in the nutrient totals as part of the dietary assessment.

### 4.2. Biochemical Assessment

As part of the biochemical assessment, we examined biomarkers for folate, vitamin B6, and vitamin B12 in highly active and sedentary women. There were no significant differences between groups for any of the B-vitamin biomarkers. All of the highly active women had biomarkers within the reference ranges for plasma folate, RBC folate, plasma vitamin B6, vitamin B12, and homocysteine. Two sedentary women had transcobalamin II concentrations below the reference range of 13–244 pg/mL, suggesting poor vitamin B12 status from the biochemical assessment. When examining their dietary assessment data, neither participant reported using dietary supplements and one reported a vitamin B12 intake much lower than the group mean (2.8 µg/day). Two of the sedentary women had PLP concentrations <20 nmol/L, suggesting poor status of vitamin B6. Upon further examination of their dietary assessment data, one was non-supplementing and reported an average vitamin B6 intake of 1.35 mg/day. The other sedentary participant reported using a dietary supplement containing vitamin B6 (2 mg/day) and was consuming on average 1.54 mg/day of vitamin B6 from food. Ten of the highly active women had elevated CRP concentrations, which may be a sign of inflammation due to over training [46,47,48]. Eight of the sedentary women had elevated CRP levels as well, which may be related to environmental factors, such as stress at home or work, pollution, illness, or other factors. The more than adequate dietary intakes of folate, vitamin B6, and vitamin B12 certainly impacted the nutrient biomarkers.

Plasma homocysteine concentrations, a functional biomarker of B-vitamin status, were within the normal range in our highly active and sedentary women and there were no differences between the groups of women. The amount of training performed by our participants did not impact plasma homocysteine concentrations. Similarly, the adequate dietary intakes for folate, vitamin B6, and vitamin B12 may have influenced plasma homocysteine concentrations. Some studies have documented acute increases in homocysteine after exercise, but then a return to baseline after a recovery or resting period. For instance, Wright et al. reported homocysteine concentrations in men increased immediately after a 30 minute bicycle ride, but began to decrease within 30 min after exercise [49]. In another study involving winter athletes, plasma homocysteine concentrations were higher during training and competition compared to baseline [50]. Dehydration and decreased blood volume after strenuous exercise may be a factor in these study results. Similarly, Gelecek and colleagues reported increases in homocysteine concentrations from baseline after one exercise session and a 6 week exercise training program [51]. These results can also be a factor of dehydration. Our study documented biomarkers for folate, vitamin B6, and vitamin B12 within the reference range for the highly active women, but we did not examine the effect of acute physical activity on these parameters.

### 4.3. Limitations

The first limitation is related to the self-reported food and activity logs. Participants were instructed how to complete the forms and provided written information to use as a guide. However, many food records needed clarification of contents, amounts consumed, and preparation. Participants may have omitted some of their dietary intake to make it look as though they consumed less. Examples include omitting “unhealthy” foods and reporting intake of more socially desirable foods. The second limitation is that the results of this study are not applicable to all female athletes as we did not have representatives from every sport discipline. Due to the small sample sizes included in our study (basketball, *n* = 1; cross country/long-distance running, *n* = 3; gymnastics, *n* = 1; ice hockey, *n* = 1; softball, *n* = 3; swimming, *n* = 11; tennis, *n* = 4; volleyball, *n* = 5), our results may not be generalizable to female athletes participating in these same sports. A third limitation is the classification of activity level using self-report of programmed physical activity. For instance, the sedentary women could have reported engaging in <2 h of programmed physical activity but have jobs that require them to stand, walk, and complete physical movement throughout the day (i.e., childcare, retail sales, landscaping), thus confounding the results. Fourth, due to limited research, power calculations were not computed for reported vitamin B12 dietary intakes and B-vitamin biomarkers. The study was not powered to detect differences between the active and sedentary women in reported dietary folate intakes and may not have been sufficiently powered to detect differences in the other study outcome measures. This low statistical power reduces the chance of detecting any true differences between the study groups and increases the likelihood of making a type II error. Thus, the study results should be interpreted with caution and may not be generalizable to other groups of highly active and sedentary women. Fifth, selection bias may have influenced the study results. Because the participants volunteered for this nutrition study, they may be more interested in health and nutrition than their peers and may not appropriately represent other highly active and sedentary young women. Another limitation is whether the participants followed the parameters of the fasting blood draw (8-h fasting). Participants were asked the time when food/meal was consumed before the blood draw. There is also the possibility the participants did not refrain from physical activity or smoking (if they reported social smoking) for 48 h prior to the blood draw. Acute bouts of strenuous exercise may have an impact on CRP levels and smoking may alter folate status and CRP concentrations [46,47,48,52].

### 4.4. Future Studies

This study is one of few to comprehensively assess B-vitamin status (folate, vitamin B6, and vitamin B12) using both dietary (food, dietary supplements) and biochemical assessments in highly active and sedentary women. We did not report losses of the B-vitamins or complete a clinical exam looking for physical signs and symptoms related to B-vitamin status. Additional biomarkers (both static and functional) could further describe B-vitamin status of highly active women. Future studies should also incorporate larger sample sizes representing athletes from varied sports.

## 5. Conclusions

This study described a comprehensive nutrition assessment of the B-vitamins in highly active and sedentary women. Although the highly active women had significantly higher dietary intakes of energy, folate, vitamin B6, and vitamin B12, there were no significant differences between groups for the biomarkers of B-vitamin status. The dietary intakes reported by the women in this study were much higher than the dietary intakes reported in the research literature. Additionally, 5 women (4 highly active, 1 sedentary) exceeded the UL for folate with the consumption of fortified foods. In this study, all of the women that used dietary supplements met the 1998 RDA for folate, vitamin B6, and vitamin B12. However, the UL for folate was exceeded by 5 highly active and 8 sedentary women when the intake included both food and supplements. Furthermore, all of the highly active women had biomarkers of B-vitamin status within the reference ranges, reflecting the adequate dietary intakes (food, dietary supplements) for folate, vitamin B6, and vitamin B12.

## Figures and Tables

**Table 1:** Descriptive characteristics of highly active and sedentary women ^a^.

Characteristics	Activity Level		*p*-Value
	Highly Active	Sedentary	
	*n* = 29	*n* = 29	
**Descriptives**			
Age (years)	20 ± 2	24 ± 3	<0.01 **
Height (cm)	169 ± 7	166 ± 8	0.11
Weight (kg)	68 ± 9	62 ± 10	0.03 *
Body mass index (kg/m^2^)	23.8 ± 3.5	22.6 ± 3.0	0.17
Programmed physical activity (min/day)	169 ± 241	6 ± 8	
**Energy intake**			
Total energy (kcal/day)	2373 ± 616	1820 ± 403	<0.01 **
Relative energy (kcal/kg body weight)	35.2 ± 8.9	29.6 ± 7.2	0.01 *
**Energy expenditure**			
Estimated energy requirement (EER) (kcal)	2350 ± 168	1972 ± 132	<0.01 **
Energy intake/EER (%)	101 ± 25	92 ± 19	0.15
**Race/ethnicity** ^b^			
African American (*n* (%))	0 (0)	0 (0)	
Asian/Pacific Islander (*n* (%))	1 (3)	0 (0)	
Native American (*n* (%))	0 (0)	1 (4)	
Caucasian (not of Hispanic origin) (*n* (%))	20 (69)	23 (82)	
Hispanic (*n* (%))	8 (28)	4 (14)	
**Sport**			
Basketball (*n* (%))	1 (3)		
Cross country/Long distance running (*n* (%))	3 (10)		
Gymnastics (*n* (%))	1 (3)		
Ice hockey (*n* (%))	1 (3)		
Softball (*n* (%))	3 (10)		
Swimming (*n* (%))	11 (38)		
Tennis (*n* (%))	4 (11)		
Volleyball (*n* (%))	5 (17)		

^a^ Values are reported as mean ± standard deviation, except where noted; ^b^ One sedentary participant did not provide this information; * *p* < 0.05; ** *p* < 0.01.

**Table 2:** Folate intakes (food and dietary supplements) in highly active and sedentary women ^a^.

Intake Variable ^b^	Activity Level		*p*-Value
	Highly Active	Sedentary	
	*n* = 29	*n* = 29	
**Folate intake from food**			
Folate (natural) (μg/day)	284 ± 119	190 ± 75	<0.01 **
	256 (175)	190 (116)	
Folic acid (fortified foods) (μg/day)	345 ± 213	238 ± 140	0.03 *
	302 (272)	197 (155)	
Folate (natural + fortified foods) (μg/day DFE) ^c,d^	867 ± 391	595 ± 250	<0.01 **
	777 (520)	537 (344)	
Folate density (μg DFE/1000 kcal) ^c,e^	364 ± 135	336 ± 154	0.47
	325 (205)	288 (132)	
**Supplement contribution for those that reported supplement use**			
Participants that reported folate dietary supplement use (*n* (%))	9 (31)	12 (41)	
Folic acid (dietary supplements) (μg/day)	564 ± 272	935 ± 438	0.04 *
	588 (364)	680 (595)	
Folate (natural) + folic acid (fortified foods + dietary supplements) (μg/day DFE) ^c,d^	1470 ± 672	1468 ± 473	0.99
	1232 (941)	1447 (670)	
Folate (natural) + folic acid (fortified foods + dietary supplements) density (μg DFE/1000 kcal) ^c,e^	621 ± 299	904 ± 365	0.07
	509 (358)	775 (508)	

^a^ Values expressed as mean ± standard deviation and median (interquartile range), except where noted. ^b^ Intake variable determined by 7-day weighed food records analyzed using the United States Department of Agriculture (USDA) National Nutrient Database for Standard Reference, Release 20. ^c^ μg/day of DFE (Dietary Folate Equivalents) = (folic acid × 1.7) + natural food folate. ^d^ Folate Recommended Dietary Allowance (RDA) for women ages 14–18 = 400 μg/day of DFE. Folate RDA for women ages 19–50 = 400 μg/day of DFE. ^e^ Recommended value for DFE density: 250 μg of DFE/1000 kcals [44]. * *p* < 0.05. ** *p* < 0.01.

**Table 3:** Vitamin B6 intakes (food and dietary supplements) in highly active and sedentary women ^a^.

Intake Variable ^b^	Activity Level		*p*-Value
	Highly Active	Sedentary	
	*n* = 29	*n* = 29	
**Vitamin B6 intake from food**			
Vitamin B6 (mg/day) ^c^	3.5 ± 2.2	1.8 ± 0.7	<0.01 **
	2.8 (1.9)	1.6 (0.8)	
Vitamin B6 density (mg/1000 kcal) ^d^	1.6 ± 1.2	1.0 ± 0.4	0.03 *
	1.1 (0.8)	0.9 (0.5)	
**Supplement contribution for those that reported supplement use**			
Participants that reported vitamin B6 dietary supplement use (*n* (%))	8 (28)	12 (41)	
Vitamin B6 (dietary supplements) (mg/day)	7.6 ± 9.2	14.0 ± 29.4	0.56
	2.7 (14.0)	2.3 (3.0)	
Vitamin B6 (food) + vitamin B6 (dietary supplements) (mg/day) ^c^	11.5 ± 9.8	15.8 ± 29.3	0.70
	7.6 (15.4)	4.4 (3.2)	
Vitamin B6 (food) + vitamin B6 (dietary supplements) density (mg/1000 kcal) ^d^	5.0 ± 4.1	9.0 ± 15.3	0.49
	3.4 (7.9)	2.5 (3.6)	

^a^ Values expressed as mean ± standard deviation and median (interquartile range), except where noted. ^b^ Intake variable determined by 7-day weighed food records analyzed using the United States Department of Agriculture (USDA) National Nutrient Database for Standard Reference, Release 20. ^c^ RDA for vitamin B6 for girls ages 14–18 = 1.2 mg/day. RDA for vitamin B6 for women ages 19–50 = 1.3 mg/day. ^d^ Recommended value for vitamin B6 density: 1 mg/1000 kcals [44]. * *p* < 0.05. ** *p* < 0.01.

**Table 4:** Vitamin B12 intakes (food and dietary supplements) in highly active and sedentary women ^a^.

Intake Variable ^b^	Activity Level		*p*-Value
	Highly Active	Sedentary	
	*n* = 29	*n* = 29	
**Vitamin B12 intake from food**			
Synthetic vitamin B12 (μg/day)	3.8 ± 5.8	1.6 ± 2.2	0.05
	2.7(1.6)	0.7(1.8)	
Vitamin B12 (μg/day) ^c^	8.1 ± 6.3	4.7 ± 2.4	<0.01 **
	6.1(5.4)	4.3(2.0)	
Vitamin B12 density (μg/1000 kcal) ^d^	3.6 ± 3.7	2.7 ± 1.5	0.21
	2.9(1.3)	2.2(1.5)	
**Supplement contribution for those that reported supplement use**			
Participants that reported vitamin B12 dietary supplement use (*n*(%))	9 (31)	12 (41)	
Vitamin B12 (dietary supplements) (μg/day)	34.8 ± 63.5	36.4 ± 73.8	0.96
	10.3(33.0)	7.0(21.0)	
Vitamin B12 (food) + vitamin B12 (dietary supplements) (μg/day) ^c^	38.3 ± 66.0	37.6 ± 74.0	0.98
	10.4(35.6)	9.1(21.6)	
Vitamin B12 (food) + vitamin B12 (dietary supplements) density (μg/1000 kcal) ^d^	15.9 ± 25.6	23.7 ± 48.4	0.66
	6.5(16.2)	5.6(15.8)	

^a^ Values expressed as mean ± standard deviation and median (interquartile range), except where noted. ^b^ Intake variable determined by 7-day weighed food records analyzed using the United States Department of Agriculture (USDA) National Nutrient Database for Standard Reference, Release 20. ^c^ RDA for vitamin B12 for girls ages 14–18 = 2.4 µg/day. RDA for vitamin B12 for women ages 19–50 = 2.4 μg/day. Values include natural vitamin B12 and synthetic vitamin B12 added to food. ^d^ Recommended value for vitamin B12 density: 1.5 μg/1000 kcals [44]. * *p* < 0.05. ** *p* < 0.01.

**Table 5:** Evaluation of nutrient adequacy from food using DRI ^a^ recommendations of highly active and sedentary women.

Nutrient/DRI Factors	Activity Level		Reference Values: Girls 14–18 years ^b^	Reference Values: Women 19–50 years ^b^
	Highly Active	Sedentary		
	*n* = 29	*n* = 29		
Folate				
Met EAR (*n* (%)) ^c^	28 (96)	26 (90)	330 μg/day	320 μg/day
Met RDA (*n* (%)) ^d^	26 (90)	23 (79)	400 μg/day	400 μg/day
Exceeded UL (*n* (%)) ^e,f^	4 (14)	1 (3)	800 μg/day	1000 μg/day
Vitamin B6				
Met EAR (*n* (%)) ^c^	29 (100)	26 (90)	1.0 mg/day	1.1 mg/day
Met RDA (*n* (%)) ^d^	27 (93)	25 (86)	1.2 mg/day	1.3 mg/day
Exceeded UL (*n* (%)) ^e^	0 (0)	0 (0)	80 mg/day	100 mg/day
Vitamin B12				
Met EAR (*n* (%)) ^c^	29 (100)	29 (100)	2.0 μg/day	2.0 μg/day
Met RDA (*n* (%)) ^d^	29 (100)	27 (93)	2.4 μg/day	2.4 μg/day
Exceeded UL (*n* (%)) ^e,g^	--	--	--	--

^a^ DRI = Dietary Reference Intakes. ^b^ Reference value from Food and Nutrition Board, Institute of Medicine [18]. ^c^ EAR = Estimated Average Requirement. ^d^ RDA = Recommended Daily Allowance. ^e^ UL = Tolerable Upper Intake Level. ^f^ Because the UL for folate applies to synthetic forms (fortified foods, dietary supplements), this assessment only includes the contribution from fortified foods. ^g^ A UL for vitamin B12 has not been established.

**Table 6:** Evaluation of nutrient adequacy from food and supplements using the DRI ^a^ recommendations for the highly active and sedentary women that reported dietary supplement use.

Nutrient/DRI Factors	Activity Level		Reference Values: Girls 14–18 Yeras ^b^	Reference Values: Women 19–50 Years ^b^
	Highly Active	Sedentary		
Folate				
Participants that reported folate dietary supplement use	*n* = 9	*n* = 12		
Met EAR (*n* (%)) ^c^	9 (100)	12 (100)	330 μg/day	320 μg/day
Met RDA (*n* (%)) ^d^	9 (100)	12 (100)	400 μg/day	400 μg/day
Exceeded UL (*n* (%)) ^e,f^	5 (56)	8 (67)	800 μg/day	1000 μg/day
Vitamin B6				
Participants that reported vitamin B6 dietary supplement use	*n* = 8	*n* = 12		
Met EAR (*n* (%)) ^c^	8 (100)	12 (100)	1.0 mg/day	1.1 mg/day
Met RDA (*n* (%)) ^d^	8 (100)	12 (100)	1.2 mg/day	1.3 mg/day
Exceeded UL (*n* (%)) ^e^	0 (0)	1 (8)	80 mg/day	100 mg/day
Vitamin B12				
Participants that reported vitamin B12 dietary supplement use	*n* = 9	*n* = 12		
Met EAR (*n* (%)) ^c^	9 (100)	12 (100)	2.0 μg/day	2.0 μg/day
Met RDA (*n* (%)) ^d^	9 (100)	12 (100)	2.4 μg/day	2.4 μg/day
Exceeded UL (*n* (%)) ^e,g^	--	--	--	--

^a^ DRI—Dietary Reference Intakes. ^b^ Reference value from Food and Nutrition Board, Institute of Medicine [18]. ^c^ EAR—Estimated Average Requirement. ^d^ RDA—Recommended Daily Allowance. ^e^ UL—Tolerable Upper Intake Level. ^f^ Because the UL for folate applies to synthetic forms (fortified foods, dietary supplements), this assessment only includes the contribution from fortified foods and dietary supplements. ^g^ A UL for vitamin B12 has not been established.

**Table 7:** Biochemical markers of highly active and sedentary women ^a,b^.

Blood Parameter	Reference Range	Activity Level		*p*-Value
		Highly Active	Sedentary	
		*n* = 29	*n* = 29	
Folate ^c^	>3 ng/mL	11 ± 4	11 ± 4	0.91
Number below reference range (*n* (%))		0 (0)	0 (0)	
Red blood cell folate ^c^	>140 ng/mL	444 ± 83	436 ± 122	0.79
Number below reference range (*n* (%))		0 (0)	0 (0)	
Vitamin B12 ^c^	>170 pg/mL	647 ± 267	552 ± 168	0.11
Number below reference range (*n* (%))		0 (0)	0 (0)	
Transcobalamin II ^d^	13–244 pg/mL	148 ± 115	146 ± 82	0.92
Number below reference range (*n* (%))		0 (0)	2 (7)	
Number above reference range (*n* (%))		2 (7)	2 (7)	
Pyridoxal 5′-phosphate ^c^	>20 nmol/L	53 ± 34	45 ± 26	0.33
Number below reference range (*n* (%))		0 (0)	2 (7)	
Homocysteine ^c^	<14 μmol/L	6 ± 2	6 ± 2	0.93
Number above reference range (*n* (%))		0 (0)	0 (0)	
Mean corpuscular volume ^e^	78–100 fL	89 ± 6	89 ± 3	0.91
Participants below the reference range (*n* (%))		1 (3)	0 (0)	
Hemoglobin ^e^	11.5–16.0 g/dL	13.5 ± 1.1	13.8 ± 0.7	0.23
Participants below the reference range (*n* (%))		1 (3)	0 (0)	
Hematocrit ^e^	35%–48%	40 ± 3	40 ± 2	0.78
Participants below the reference range (*n* (%))		1 (3)	0 (0)	
High sensitivity C-reactive protein ^e^ median (interquartile range)	<1.0 mg/L	0.5 (3.2)	0.4 (1.0)	0.55
Participants above the reference range (*n* (%))		10 (34)	8 (28)	

^a^ Mean ± standard deviation, except where noted. ^b^ Independent sample t-tests used to examine differences for all parameters except C-reactive protein (Mann Whitney U test). ^c^ Reference value from Food and Nutrition Board, Institute of Medicine [18]. ^d^ Reference value from Herzlich and Herbert [37]. ^e^ Reference value from Sonora Quest Laboratories. * *p* < 0.05.
